# Epidemiology and current treatment patterns of treatment-resistant depression in Scotland: a CPRD study

**DOI:** 10.1192/bjo.2021.876

**Published:** 2021-06-18

**Authors:** Timothy Ming, Tom Denee, Gemma Scott, Joachim Morrens, Christopher Weatherburn

**Affiliations:** 1 Janssen-Cilag Ltd; 2 Janssen Cilag; 3 Dundee Health and Social Care Partnership

## Abstract

**Aims:**

To assess the incidence and treatments currently used in clinical practice for the treatment of treatment-resistant depression (TRD) in Scotland.

**Background:**

Patients with major depressive disorder (MDD) who have not responded to at least two successive antidepressant (AD) treatments in a single episode are described as having Treatment-Resistant Depression (TRD). Epidemiological data on TRD in Scotland is lacking. Furthermore, there is no data to our knowledge on therapies prescribed in Scottish clinical practice to treat TRD.

**Method:**

A retrospective, longitudinal cohort study was conducted using Clinical Practice Research Datalink (CPRD) medical records. Adult patients were indexed on AD prescription, requiring MDD diagnosis within 90 days, from Jan 2011-May 2018 with 360-day baseline and 180-day minimum follow-up periods. Failure of ≥2 adequate oral AD regimens following indexing constituted TRD classification. Incidence rates of MDD and TRD (within the MDD cohort) and treatment lines following TRD classification were derived.

**Result:**

The analysis included 20,059 patients with MDD (mean age 44 years, 63% female, median follow-up 59 months); 1,374 (6.8%) were classified as TRD. Median time-to-TRD classification was 25 months. The incidence rate of MDD was 15.9 per 1,000 patient-years and for TRD was 14.7 per 1,000 MDD-patient-years. For all first four post-TRD treatment lines, SSRI monotherapy was the most commonly prescribed therapy, followed by combination (dual/triple) therapy and augmentation therapy (at least one oral AD supplemented with lithium, an antipsychotic or an anticonvulsant therapy). At first-line of TRD treatment, 1,050 (76.4%) patients received monotherapy AD, 212 (15.4%) received combination AD therapy and 112 (8.2%) received augmentation therapy. The most common monotherapy treatments at first-line TRD were sertraline (15.6%), mirtazapine (13.8%), fluoxetine (12.2%) and venlafaxine (11.6%). Among combination therapies, mirtazapine, venlafaxine, sertraline and amitriptyline were frequently used. Among the TRD and MDD cohort, no somatic treatments were coded in CPRD, although the use of these treatments was likely underestimated.

**Conclusion:**

Monotherapy AD treatment was the most common therapy type for all four post-TRD treatment lines. These data support the need for new treatments that can achieve and maintain therapeutic response, and avoid continuous cycling through similar AD therapies.

This study was sponsored by Janssen Cilag Ltd.

